# One Hundred Consecutive Cases of Skin Disease, IV

**Published:** 1891-04

**Authors:** M. B. Hutchins

**Affiliations:** Lecturer on Diseases of the Skin, Atlanta Medical College


### ONE HUNDRED CONSECUTIVE CASES OF SKIN DISEASE.

### IV.

BY M. B. HUTCHINS, M. D.,
Lecturer on Diseases of the Skin, Atlanta Medical College.

### SEBORRHOIC ECZEMA.

In the March, 1889, number of THE JOURNAL I gave an abstract of the description, by Unna, of Hamburg, of this disease, the title being originated by him. It is therefore only necessary to refer the reader to that description and thus save repetition. Unna's view does not now appear to have been so generally adopted by dermatologists as was expected at that time, but no matter what we call the condition, the fact remains that Unna's treatment is good enough, if not the best, for it.

The first case which I saw in Atlanta was that of an infant aged four months. Old seborrhœa, closely adherent flakes of dirty, greasy, though rather dry, sebaceous matter on scalp, with thickening, redness and scaling of skin of temples and cheeks; fissuring behind ears and slightly on other diseased parts. The parents believed the disease would be *driven back into the blood* by local treatment, and as that treatment was chiefly indicated, I did nothing for the case.

Manifestations of this disease varied from simple dandruff, with a few reddened, scaly points, with itching when warm, to large patches on scalp and other parts, sero-crusted and discharging. The majority corresponded to the condition yet generally described as seborrhœa of the type "oleosa;" greasy scales on scalp and an oily condition of face, especially of nose and naso-labial furrows and forehead.

A typical case of eczema seborrhoicum was that of a physician living out of the city, age thirty-five. Disease present several years. Greasy scales and flakes on scalp. Frontal baldness, progressing towards vertex. Entire skin oily, and patient perspired freely. A few faintly red patches on scalp and face. Redness and formation of greasy scales in eyebrows (which had become thin), mustache and beard. Scales, many of them, loose and occupying hairs above the skin. From clavicles to level of nipples (chest very hairy) gyrate patches of redness, sharply defined by normal skin; no thickening, a slight disquamation of greasy scales. Similar condition, modified by moisture of parts, in scroto-femoral region. Some itching in diseased skin. Got well under—

R. Resorcin, ʒi–℈i.

Sp. vini rect.,

Aquæ, aa. ℥ii.

M. Sig.—Apply thoroughly morning and night ; at night following with—

R. Resorcin, gr. xv.
Ungt. zinc oxid., ℥i.

M. Sig.—Rub well in.

The age of these patients varied from four months to forty-five years. Majority of the patients, thirteen in all, were males, seeming to indicate that the trouble is more common in this sex. But I do not think the figures would furnish reliable statistics upon this subject.

Health of the patients, as a rule, was not "bad," but it is a disease most likely to occur in persons a little "run down."

At the risk of being called empirical, perhaps justly, I confess that I have "stuck to" the resorcin, average three per cent., in lotion and ointment, beginning the treatment of the scalp with a thorough cleansing with plain soap and water, repeating this once or twice per week. Two months' treatment, average, should cure most cases, but of course the dandruff may return some day under the influence of conditions of the system which lower vitality. As preventive of return, the following, applied occasionally, will be found useful:

R. Resorcin, gr. xv.
Lanolin pulv., gr. x.
Sp. vini rect.,
Aquæ, aa. ℥ss.

M.

In the above the lanolin, partially in solution, prevents the hair and scalp from being left in too dry condition.

16½ *Whitehall St.*

---

The legislature of Texas has now under consideration a bill to regulate the practice of medicine, and to create a board of medical examiners in Texas, and the probability seems to be that the measure will pass. This is at the instance of the Committee on Legislation of the State Medical Association, and we congratulate our brethren of the Lone Star State.

